# Low-Cost Interrogation System for Long-Period Fiber Gratings Applied to Remote Sensing

**DOI:** 10.3390/s19071500

**Published:** 2019-03-28

**Authors:** P.S.S. dos Santos, P.A.S. Jorge, José M.M.M. de Almeida, L. Coelho

**Affiliations:** 1 INESC TEC—Institute for Systems and Computer Engineering, Technology and Science, 4200-465 Porto, Portugal; pedro.jorge@inesctec.pt (P.A.S.J.); jmmma@utad.pt (J.M.M.M.d.A.); 2 Department of Physics and Astronomy of Faculty of Sciences, University of Porto, 4169-007 Porto, Portugal; 3 Department of Physics, School of Sciences and Technology, University of Trás-os-Montes e Alto Douro, 5001-801 Vila Real, Portugal

**Keywords:** long period fiber grating, interrogation system, corrosion sensing, remote sensing, optical fiber sensor

## Abstract

We present a portable and low-cost system for interrogation of long-period fiber gratings (LPFGs) costing around a 30th of the price of a typical setup using an optical spectrum analyzer and a broadband light source. The unit is capable of performing real-time monitoring or as a stand-alone data-logger. The proposed technique uses three thermally modulated fiber-coupled laser diodes, sweeping a few nanometers around their central wavelength. The light signal is then modulated by the LPFG and its intensity is acquired by a single photo-detector. Through curve-fitting algorithms the sensor transmission spectrum is reconstructed. Testing and validation were accomplished by inducing variations in the spectral features of an LPFG through changes either in external air temperature from 22 to 425 °C or in refractive index (RI) of the surrounding medium from 1.3000 to 1.4240. A dynamic resolution between 3.5 and 1.9 °C was achieved, in temperatures from 125 to 325 °C. In RI measurements, maximum wavelength and optical power deviations of 2.75 nm and 2.86 dB, respectively, were obtained in the range from 1530 to 1570 nm. The worse RI resolution obtained was $3.47\times 10^{-3}$. The interrogation platform was then applied in the detection of iron corrosion, expressing wavelength peak values within 1.12 nm from the real value in the region between 1530 and 1570 nm.

## 1. Introduction

Optical fiber sensors are resistant to most common chemicals, lightweight, inherently small, and capable of withstanding high temperatures, and possess immunity to electromagnetic interference. These characteristics together with their flexible geometry make them promising solutions for corrosion detection in remote and hard-to-reach areas, including sea-based metallic infrastructures [1].

Sensors based on long-period fiber gratings (LPFGs) have been used in the determination of several chemical and physical parameters in multiple harsh environments [2,3,4]. This type of sensors makes use of light coupling between the fundamental core mode with co-propagating cladding modes. Their spectral behavior consists of high attenuation rejection bands [5].

The fabrication of LPFGs is done by applying a periodic modulation on the core refractive index (RI), which can be accomplished using phase masks or point-by-point markings, with femtosecond lasers, CO_2_ lasers, or induced electric-arc discharges in the fiber [6,7]. The spectral position of their attenuation bands depends on the RI of the surrounding medium [8].

Further deposition of thin films around the surface can greatly change the effective RI of the cladding, enhancing its sensitivity to specific phenomena [4]. The biggest challenge of using LPFGs arises from the employed interrogation techniques, and although various systems have been used, most of them require the use of stable broadband light sources (BBLS) and optical spectrum analyzers (OSAs), which makes the system bulky and expensive, ideal for laboratory environment but not suitable for field applications.

Other LPFGs interrogation techniques can be employed, such as that proposed by Bhatia [2] in a temperature sensor, which used one laser at a specific wavelength coinciding with one side of a LPFG peak. This methodology lacked a reference point to know on which side of the grating the peak was situated and also does not account for changes in amplitude and widening of the interrogated peak. A displacement sensor insensitive to temperature using two fiber loop mirrors with LPFG on each mirror was used as a displacement sensor using an Optical Time-Doman Reflectometer (OTDR), but since this scheme only uses the LPFG amplitude changes, it is not suitable for sensing several parameters, such as refractive index, since it is usual to perform measurements in terms of wavelength shift [9].

Allsop et al. [10] demonstrated a LPFG interrogated with two modulated fiber Bragg grating (FBG) at each side of an LPFG using a derivative spectroscopy technique applied on curvature sensing, but this required the use of refractive index insensitive LPFGs and embedding them in low (or negative) thermal expansion substrates to compensate for temperature fluctuations, increasing the overall cost of the setup. Furthermore, this technique was only capable of measuring curvature. Techniques using two modulated FBGs were also proposed by Carvalho et al. [11] however, the need for a broadband light source, LPFG mirrored with silver nitrate, erbium-doped fiber amplification, Raman laser, wavelength division multiplexers (WDM), and at least two optical circulators does not make it the potentially low-cost setup intended in this manuscript.

Interrogation using a mechanically scannable arrayed wave-guide grating (AWG) has been proposed based on space-to-wavelength mapping by mechanically scanning the input light beam along the input coupler facet of an AWG through a sampled chirped FBG with multiple peaks [12]. A similar AWG-based technique using a tunable laser source was suggested by Xiao et al. [13]. Despite its sub-picometer resolution, the cost of the AWG and the need for setup duplication if another LPFG needs to be used as reference can disfavor its implementation where a cost-effective method is desirable.

Commercial optical interrogators with embedded software are available to perform interrogation of FBGs at lower cost and are smaller than the typical OSA and BBLS setup. Although specifically designed to interrogate FBG-based sensors, they can be adapted to interrogate LPFGs but usually present picometer and even sub-picometer resolution, which is more than necessary for analyzing the broader band characteristic of an LPFG. Consequently, other interrogators can be fabricated for a lower cost than the price of related commercial options.

Overcoming these difficulties can be achieved by developing an interrogation unit for in-field implementation, with portability, low-cost, low-power, and data-logging capabilities, and the ability of withstanding harsh environments. This proposal gains relevance when remote and long acquisition time sensing is required. One example of this phenomena is when a corrosion-sensing mechanism is employed, especially because this process in metallic structures takes several steps before the need for structural repair. These processes are monitored using well-established techniques. However, they can be monitored at early stages with optical sensors, thus reducing maintenance costs and potential structural failure [14,15,16].

Previous work has been done with two unmodulated DFB lasers set on both sides of an LPFG band through optical power variations at their wavelengths, which achieved high correlation with changes in multi-parameter sensing [17].

In this manuscript we present a new and affordable interrogation unit (IU) as a complete embedded solution for in-field operation, lowering the costs to around a 30th of the price of a typical setup with an OSA and BBLS. The IU is supported by a novel interrogation technique based on thermal modulation of a set of laser diodes, performing wavelength scans around their central positions. Further acquisition is performed by a single photo-detector, and through curve-fitting techniques, the desired sensing parameters are recovered.

## 2. Interrogation Technique

**Figure 1:**
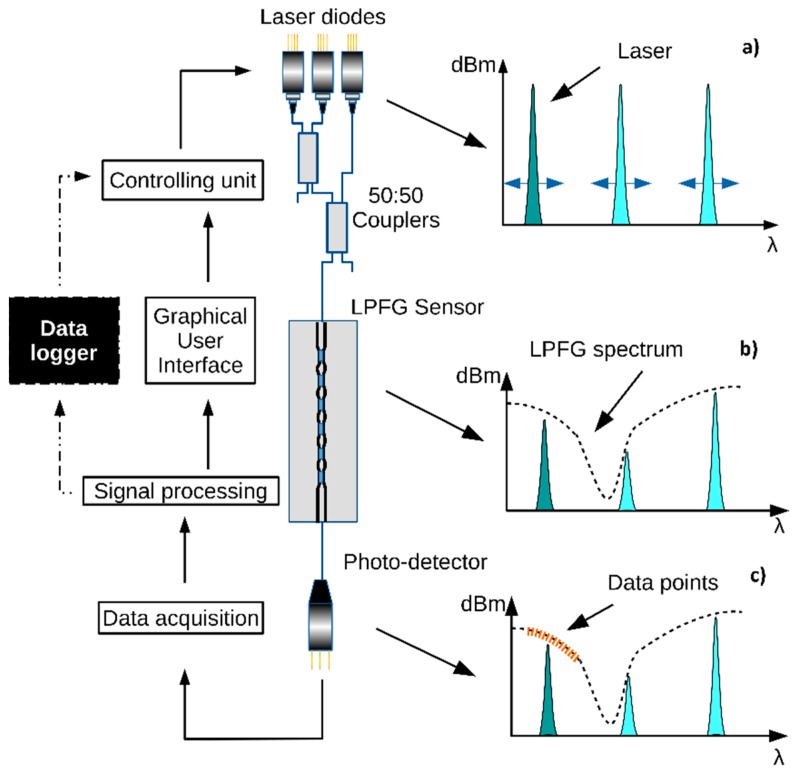
Interrogation methodology description and signal-flow. (**a**) Laser diodes being thermally swept. (**b**) Long-period fiber grating (LPFG) spectrum changing the transmitted optical power. (**c**) Data acquisition by the photo-detector for the laser diode that was turned ON.

The proposed interrogation technique is presented in Figure 1. It involves the use of three fiber coupled distributed feedback (DFB) lasers, which are thermally modulated with a Peltier element to induce a wavelength shift around their central positions. The lasers’ temperature is controlled by a proportional-integral-derivative controller (PID) algorithm [18]. A modification was added to allow going above or under the ambient temperature, switching the current direction for the Peltier, along with different PID constants. A calibration routine was performed to set a correspondence between the temperature and wavelength position of the lasers at each time instant. Since temperature changes also induce optical power variations, compensation is achieved by constantly measuring the internal photo-detectors of the DFB lasers.

The signals from the DFB lasers are then coupled together via two 50:50 optical couplers and sent to the LPFG sensor, modeling the intensity of the transmitted signal accordingly.

Only one of the DFB lasers is turned ON at a given moment, ensuring a direct match between the signal and its wavelength position. The light signal that crosses the LPFG is then measured by a photo-detector. This process is repeated with the DFB lasers at different temperatures, to maximize the swept wavelength range. The gathered data from all the photo-detector acquisitions is then processed by a software routine, recording the optical power value and mapping it onto a wavelength value.

The acquisition process can be controlled via the developed graphical user interface (GUI), where the operator can fully control the lasers’ positions and choose the necessary number of iterations, as a full temperature range is not always needed to obtain the necessary data points to construct the grating spectrum. At the end of data acquisition, Lorentzian, Voight, and Gaussian curves are fitted through several iterations by proper numerical methods and their fitting parameters coefficients are compared. Optimized curve fitting procedures were developed, taking into account the slope of the data points, and previously fitting curves characteristics, such as amplitude and Full Width at Half Maximum (FWHM). The fitting curve is then chosen by maximizing its correlation value.

**Figure 2:**
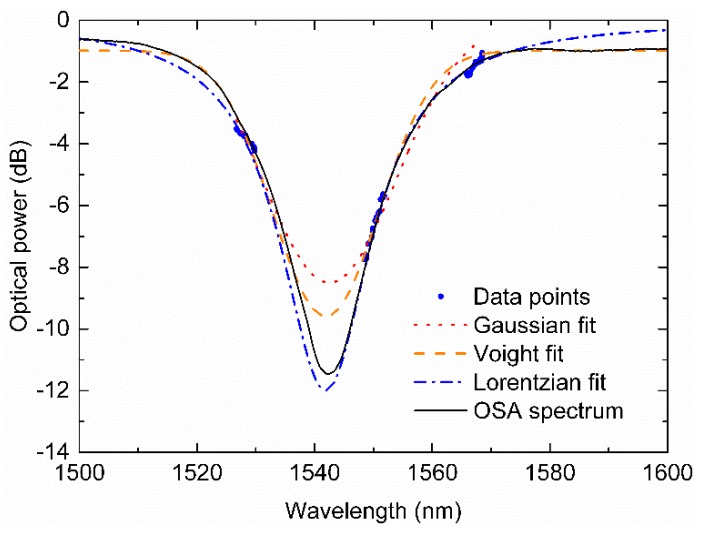
Comparison between several fitting curves for the acquired data points using the developed interrogation unit (IU) and, for validation purposes, the real LPFG spectrum measured by a typical optical spectrum analyzer (OSA) and broadband light sources (BBLS) scheme.

Figure 2 displays three different fitting curves produced and, just for comparison purposes, the real spectrum obtained from the typical setup of an OSA and BBLS.

When the device is used as a data-logger, a software routine can be programmed to schedule periodic measurements and store the obtained data in the memory. The data can then be retrieved through the GUI or by a software routine. Applying the described fitting techniques to the data, the information carried by the original LPFG spectrum is regained. This operation mode of scheduled measurements is of crucial importance to manage the power consumption requirements.

The emitted optical power of each laser is monitored in real-time through the built-in photo-detector, which is used for auto-calibration of the transmitted optical power that crosses the LPFG and reaches the interrogation unit (IU) photo-detector.

The provided modulation, due to thermal inertia, presents typical speeds of around 3.0 °C/min. A measurement involves a temperature sweep in the DFB lasers. If this sweep is made from 5 to 45 °C, corresponding to a wavelength span of 4 nm, it usually takes around 15 min. This measurement speed makes the developed IU suitable for slow-changing applications, such as corrosion sensing.

**Figure 3:**
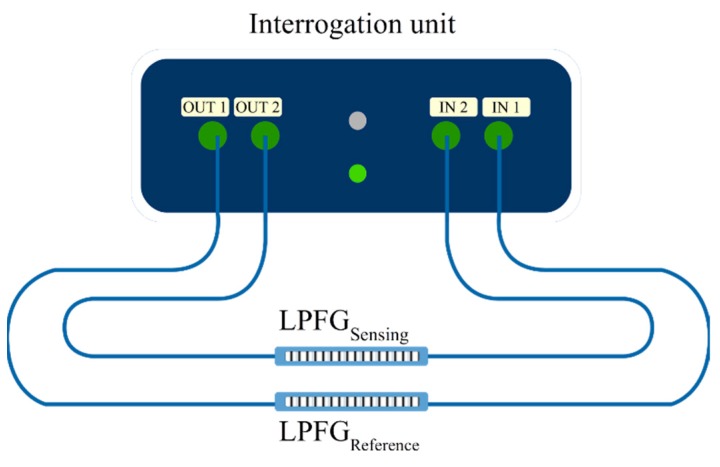
Sensing scheme with one LPFG acting as a reference sensor providing compensation to the second sensor.

**Figure 4:**
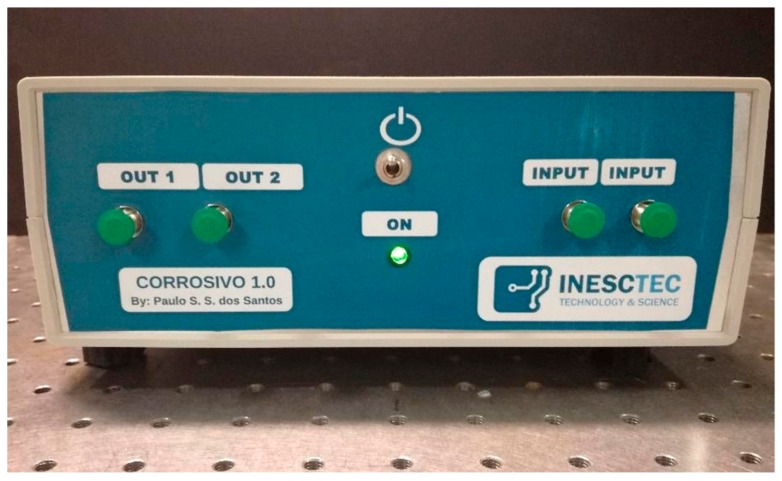
Interrogation unit prototype.

The developed IU prototype has the ability of interrogating two distinct gratings, where one can be used for the actual sensing, and the other grating can be used as a reference. Both sensors can be easily connected through two fiber connectors (FC) outputs and two FC inputs in the unit, as represented in Figure 3. Figure 4 presents the built prototype.

The IU possesses Universal Asynchronous Receive and Transmission (UART) communication, from which data can be pulled from the designed graphical user interface developed in LabVIEW. The GUI can fully control the unit in a remote location and transmit the data in real-time to a user or operate as a stand-alone data-logger. The developed unit also possesses internal batteries to perform its functions without a permanent energy source, which is recommended for long-term measurements. The energy source can be a portable source, such as a solar panel. The developed IU was also designed to minimize power consumption by entering in low-power routines when in IDLE state.

## 3. Materials and Methods

The three DFB lasers (Roithner Lasertechnik, GmbH, Vienna, Austria) used in the IU are positioned in emission wavelengths of 1530 nm, 1550 nm, and 1570 nm, respectively, with FWHM < 0.02 nm and a maximum optical power of 2 mW, except for the DFB centered at 1550 nm, which has a maximum optical power of 1 mW; thus, a compensation is made using two 50:50 couplers, with this laser occupying the position where it only passes through one coupler. To ensure an inexpensive unit design, a laser driver circuit was also developed, based on a linear constant current source, ensuring the required operating conditions, such as over-current protection, slow-start, and constant current mode. The DFB lasers were subjected to temperature and current variations to characterize their response to those parameters in terms of wavelength shift and optical power changes. Current variations were generated with the use of one laser driver (Thorlabs Diode Combi Controller ITC502, Lubeck, Germany), with a current accuracy of $3.47\times 10^{-3}$ and a ripple value lower than ±100 μA. Measurements were carried out by an OSA (Yokogawa AQ6370, Tokio, Japan). The temperature coefficients for the three DFB lasers were obtained by varying the lasers’ temperature from 5 °C to 45 °C in steps of 2 °C, using a 7.0 W Peltier module (TES1-03103T125, Roithner Lasertechnik, GmbH, Vienna, Austria). The readings were made by the OSA with 0.2-nm resolution.

**Figure 5:**
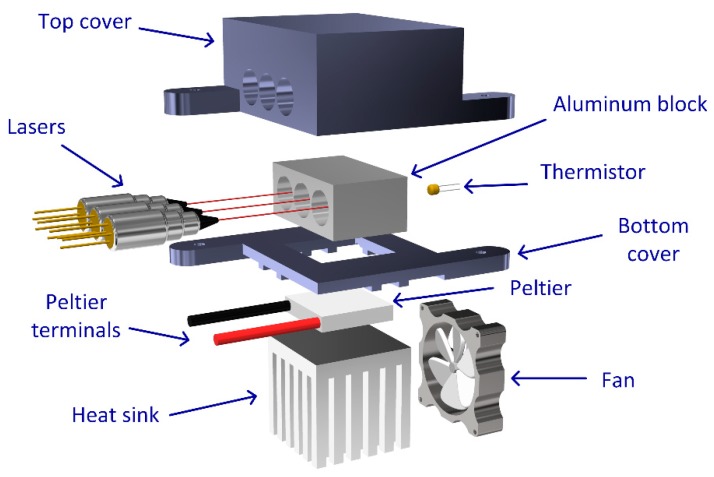
Exploded view of the components for the thermal control section.

Figure 5 shows the hardware scheme for temperature tuning of the lasers. The main structure was constructed using a 3D printed case to hold a heat sink, a Peltier element, and an aluminum block to clamp the lasers. The Peltier module is in close contact with the aluminum holder, in which the DFB lasers are embedded, providing homogeneous temperature modulation to the three lasers. The temperature readouts are made by a thermistor placed deep inside the block. The top cover is an insulation case to inhibit humidity from reaching the lasers’ enclosure and minimize heat transfer between the aluminum block and the surrounding medium. The Peltier module is completed with the aluminum heat-sink coupled with a mechanical fan with intensity adjusted in real-time.

The readouts made by the interrogating unit were done using a fiber-coupled InGaAs photo-diode (JDSU EPM 606LL, Milpitas, CA, USA). The output current from the photo-diode is obtained by a trans-impedance amplifier with adjustable gain. The voltage measurements are carried by a micro-controller that calculates the difference between these readouts and the readings from the built-in photo-detectors in the lasers.

The developed interrogation unit was then tested with LPFG fiber sensors. A set of LPFGs were inscribed in SMF28e optical fiber by electric-arc. For a grating period of 410 μm, the asymmetric 6th order cladding mode LP_1,6_ falls in the 1500–1600-nm range, with attenuations up to 25 dB. 

Temperature measurements were made with a LPFG inside a sealed oven (Termolab, Águeda, Portugal), and the temperature was changed in steps of 50±1 °C from 22 °C to 425 °C, whilst the fiber was maintained in a constant strain state, equal to the conditions upon fabrication.

Variations of spectral behavior due to RI changes were carried out by immersing the grating in a set of RI-calibrated oils (Cargille-Sacher Laboratories Inc., Cedar Grove, NJ, USA). The oil samples to be tested were placed in a V groove aligned with the sensing region and covering the entire LPFG. RI variations were conducted from 1.3000 to 1.4240, with constant strain and at room temperature.

In addition, a set of LPFGs were coated with Fe with a thickness from 10 to 50 nm around the grating region. The thin film deposition was performed by thermal evaporation of pure metal (Goodfellow, Huntingdon, England) using an electron beam evaporator (Auto 306 from Edwards Ltd., Burgess Hill, UK). The deposition chamber was kept at around $2.6\times 10^{-6}$ mbar, and it is fitted with a homemade rotary system, illustrated in Reference [19], in order to produce homogeneous coatings around the cylindrical fibers. Prior to the thin film coating, a 2-nm layer of chromium was deposited to improve the adhesion between the silica fiber and Fe thin film.

**Figure 6:**
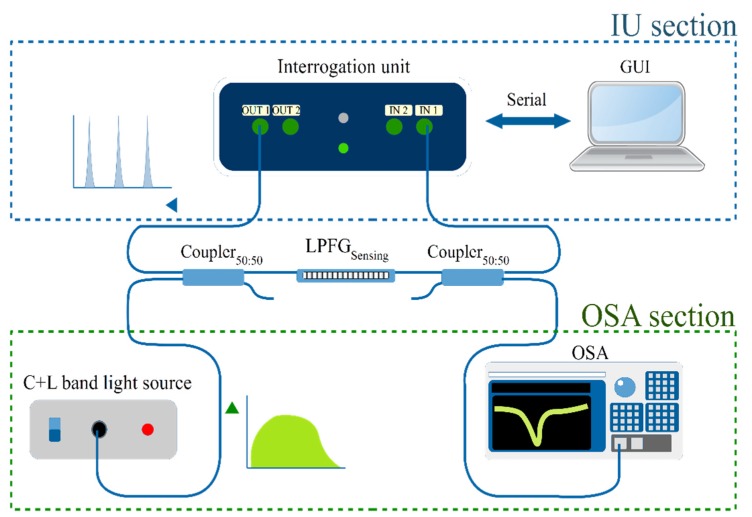
Setup for the measurements, with the LPFG as the sensing component. The graphical user interface (GUI) is used to control the interrogation unit and data acquisition. The blue section represents the IU scheme, the green section shows the BBLS/OSA utilized for system validation. Only one of the sections are making measurements at any one time.

For validation, a comparison between the developed IU scheme and a conventional setup using an OSA and a BBLS was carried. To achieve a reliable comparison between the methods, the experimental setup represented in Figure 6 was used.

The signal path for the usual OSA scheme (green section) starts in an unpolarized C + L band light source connected to the first 50:50 fiber coupler, from where the light reaches the LPFG sensor, and through the second coupler the signal is sent to the OSA. For the IU scheme (blue section), the light is supplied from the DFB lasers output and sent to the assembled LPFG through the first 50:50 coupler. The LPFG modulated signal then reaches the second coupler and is sent to the IU input photo-detector, where the software routine starts the data acquisition.

It is important to note that the IU and the BBLS/OSA schemes are never making measurements at the same time, and the OSA/BBLS is only present for system validation. For real measurement operations, the IU works with the scheme presented in Figure 3.

## 4. Results

### 4.1. DFB Lasers Characterization for Temperature and Current Modulation

**Figure 7:**
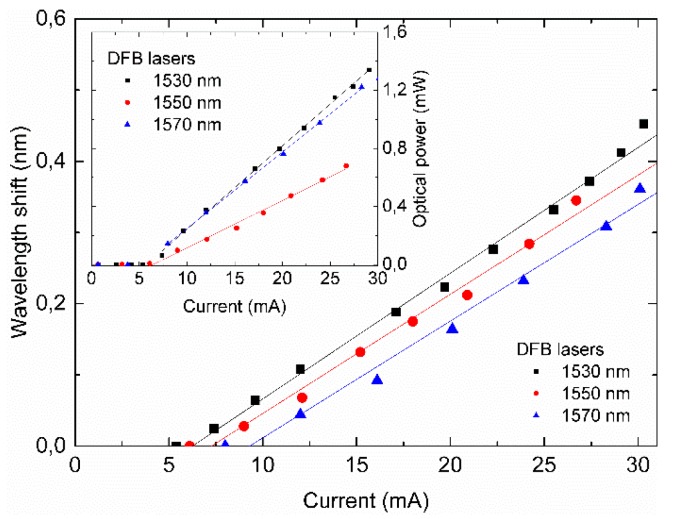
Effects of different values of the supplied current for the three laser diodes in terms of wavelength shift and optical power (inset).

The results for the wavelength and optical power response due to the current sweep are presented in Figure 7. A wavelength shift presents a linear relationship with current [20]. Sensitivity values of 17.2, 16.7, and 16.4 pm/mA were obtained, with R² values of 0.983, 0.989, and 0.987 at the DFB lasers in 1530, 1550, and 1570 nm, respectively. A linear output optical power with respect to the supplied current was also observed above the threshold current of the DFB lasers. The value of these coefficients is not enough to design any major laser tuning for the interrogation unit but enables the use of current as a fine wavelength tuning control.

**Figure 8:**
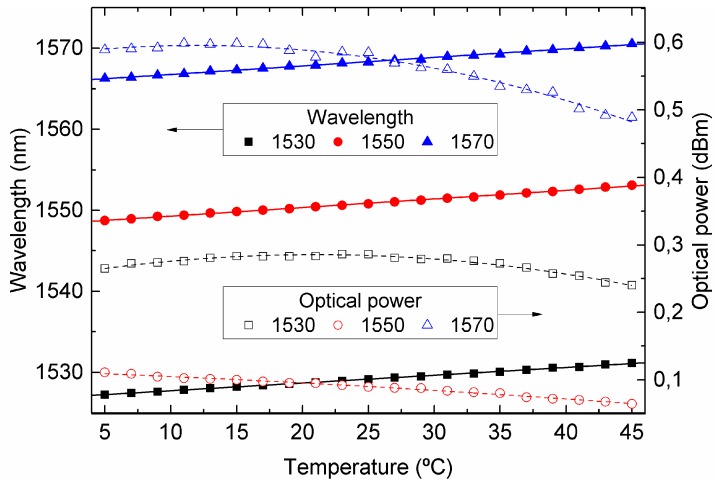
Wavelength and optical power shift for the temperature sweep of the three used DFB lasers centered at 1530, 1550, and 1570 nm, respectively.

Temperature modulation revealed a linear behavior of 105, 106, and 96 pm/°C, characterized by R² values of 0.998, 0.998, and 0.997, as can be seen in Figure 8. The DFB lasers data-sheet temperature specifications stated maximum ratings from −10 to 85 °C; thus, working below those limits will extend their life-span, and we chose a thermal tuning range from 5 to 45 °C, corresponding to a wavelength range of approximately 4 nm. The measured optical power as a function of temperature was characterized by a second-order polynomial fitting curve with R² values of 0.980, 0.992, and 0.981 [20].

Compensation of the instantaneous output optical power by each laser is performed by using their built-in photo-detectors.

### 4.2. LPFG Characterization with OSA (Temperature, Refractive Index, Curvature, Strain)

With the BBLS and OSA resolution set to 0.02 nm, temperature measurements were made with a LPFG inside a sealed oven with a resolution of ±0.1 °C, where a sensitivity of 109 pm/°C and −0.07 dB/°C as achieved. For strain and curvature sensitivities, values of 1.84 pm/με and −0.01 dB/με, and 294 nm/m^−1^ and 1.1 dB/m^−1^, respectively, were determined. The sensor sensitivity for these two parameters is important to assure low errors due to cross-measurements.

Finally, a RI sensitivity of 100 and 400 nm/RIU (RIU—refractive index unit) in the 1.3600 and 1.4200 RI region, respectively, was also obtained. This increasing sensitivity, is due to the cladding RI approach.

### 4.3. Temperature Characterization and Spectral Resolution

With the setup presented in Figure 6, the LPFG was subjected to different temperatures and its transmission spectrum was recorded in real time by the developed IU, and the acquired data were fitted to Voight, Gaussian, and Lorentzian profiles and compared with the BBLS/OSA results.

**Figure 9:**
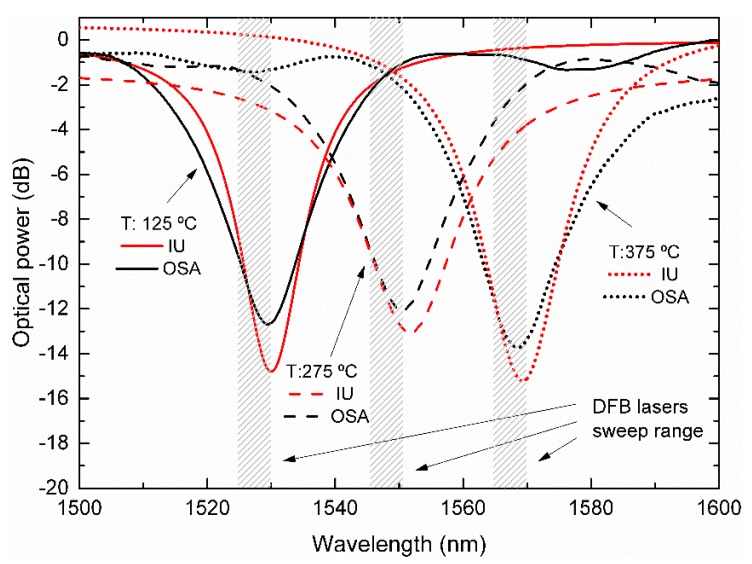
Comparison between the LPFG spectra at three different temperatures as measured by the developed IU curve fittings, with the represented spectral positions of its three DFB lasers, and the real LPFG measured by the conventional BBLS/OSA scheme.

**Figure 10:**
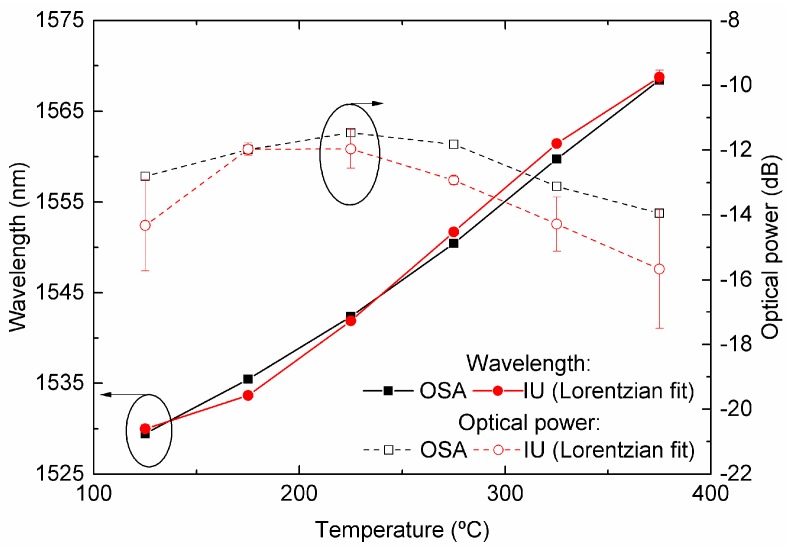
Comparison of the wavelength and optical power of the LP_16_ mode of an electric-arc produced by the LPFG subjected to various temperatures. The results obtained by the developed IU are presented in red. Shown in black, just for comparison, are the measurements obtained by a usual BBLS/OSA scheme.

Figure 9 illustrates the LPFG spectra for temperatures of 125 °C, 275 °C, and 375 °C, centered below, on, and above 1550 nm, respectively. The spectral positions of the DFB lasers are also shown. In this case, the overall best fit parameter results had an average R^2^ value of 0.997 for the complete temperature sweep. This result was obtained with the Lorentzian fit, rather than Gaussian and Voight curves, which presented average R^2^ values of 0.984 and 0.991, respectively. The comparison between the IU and OSA measurements is presented in Figure 10.

It must be observed that only points between 125 °C and 375 °C, corresponding to wavelength positions inside the range of the DFB lasers (from 1530 nm to 1570 nm) are presented. The error associated with the peak-position determination increases significantly as the peak reaches wavelengths beyond the DFB lasers’ wavelength locations, due to the lack of data points on both sides of the central position of the LPFG attenuation band.

Inside the region of interest (1530 nm to 1570 nm), the reconstructed grating peak position presented deviations between the two interrogation methods of less than 2 dB concerning the optical power. For the wavelength, the offset is either positive or negative depending on whether the rejection band is above or below 1550 nm, corresponding to the central DFB position with a maximum difference of 2.1 nm. This effect can be attributed to the asymmetry of the LPFG band produced by the inherently poor repeatability between electric-arc discharges and alignment asymmetry, producing an uneven index-step modulation in the fiber [21].

Interrogation systems based on this kind of sensors typically make use of their sensitivity in terms of wavelength shift instead of optical power changes. Therefore, in the following discussion, optical power variations will not be used as the sensing parameter.

The spectral resolution, *R,* can be estimated considering the values obtained from two measurements linked with two different temperatures, obtained with the following expression [22]:

(1) $$R=\frac{\sigma }{S_{T}}=\frac{\delta T}{\delta \lambda }\sigma$$

where *δΤ* is the amplitude of the temperature step variation, *S_T_* is the sensitivity to temperature variations, *δλ* is the difference in the wavelength resonant dip, and *σ* is the highest standard deviation between the two steps. To check the stability/repeatability of the process, a calibrated thermocouple was used in the measurements, confirming the effectiveness of this sensing system.

**Figure 11:**
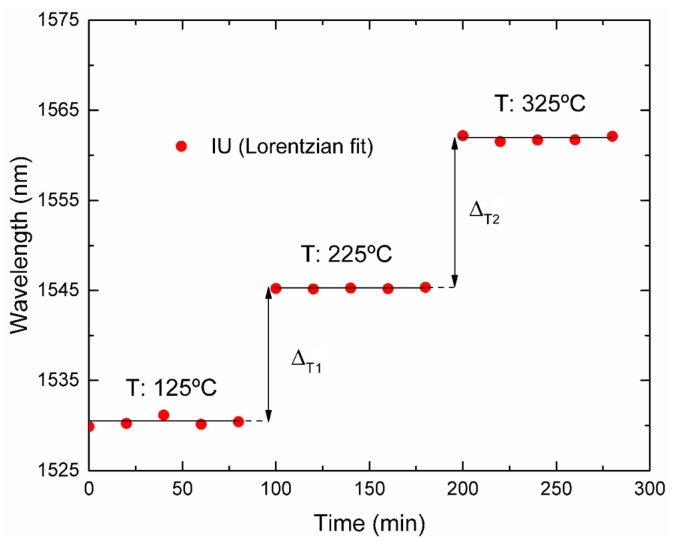
Wavelength shift of the dip of the LPFG when the temperature experiences step variations.

From the data shown in Figure 11, the spectral resolutions calculated for steps Δ_T1_ and Δ_T2_ were 3.5 °C and 1.9 °C, respectively, in the temperature range from 125 °C to 325 °C, using the developed IU. Within this range, the attenuation band falls between the DFB lasers, thus leading to an effective sampling.

On the other hand, when using the OSA and BBLS, a spectral resolution better than 1.7 °C was calculated for the same temperature steps.

### 4.4. Surrounding Refractive Index Sensing

A bare LPFG with its peak situated at 1539.56 nm in air was used to measure the effect of the surrounding RI on the attenuation band using both the OSA and the IU.

**Figure 12:**
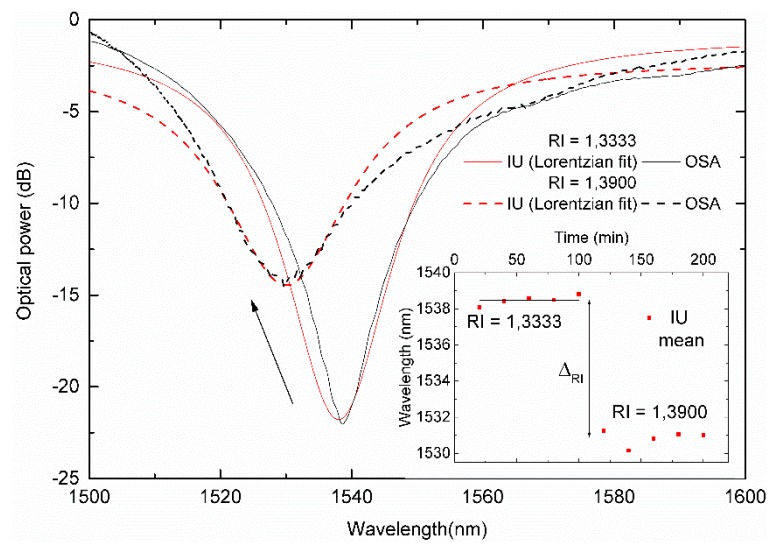
Comparison between the interrogation unit (IU) with a Lorentzian curve fit and the OSA spectra for two refractive index values. The step response is presented in the inset.

Figure 12 shows the spectra of a LPFG linked with two different RI (1.3333 and 1.3900), as measured by the IU and compared with the BBLS/OSA scheme. The spectral resolution, R, which can be estimated following the above method is presented in the inset. The Δ_RI_ has the LPFG dip located between the DFB lasers at 1530 nm and 1550 nm. The worse RI resolution obtained was $3.47\times 10^{-3}$ with the IU and $1.03\times 10^{-3}$ for the OSA.

**Figure 13:**
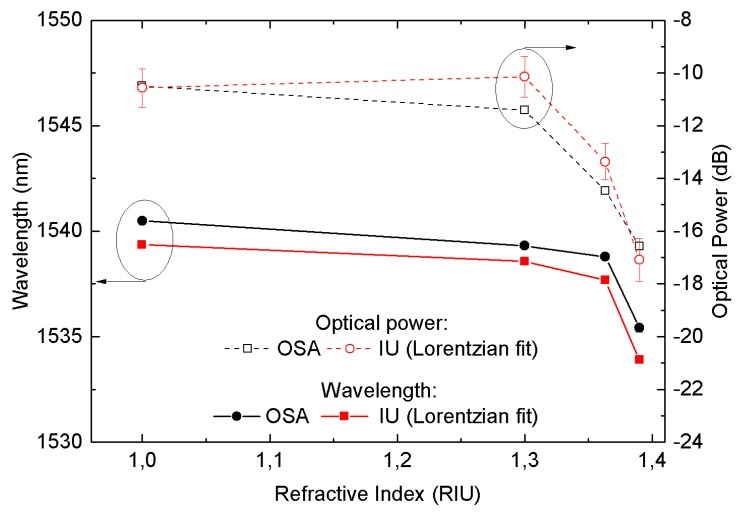
Comparison between the IU with a Lorentzian curve fit and the OSA for the refractive index response of a LPFG peak wavelength and optical power.

In this case, the overall best fit parameter results were again obtained with a Lorentzian fit. The comparison between the measured wavelength and optical power values are presented in Figure 13. A constant wavelength offset is present, as expected, due to the dip being below the central DFB laser. Nevertheless, the data follow the expected behavior, with both wavelength and optical power shift presenting the maximum values as the external RI approaches the silica RI.

### 4.5. Corrosion of Fe in Water

Previous works showed the possibility of using a Fe-coated LPFG as a corrosion sensor [19,23]. Thus, as an example of use of the developed IU, the corrosion-induced spectral characteristics variations of a 20-nm thick LPFG were followed.

As the corrosion process in salt water takes place, the amplitude of the attenuation band increases and shifts to lower wavelengths. The attenuation band of the Fe-coated LPFG was initially centered at 1550.91 nm, coinciding with one of the lasers’ central position. For this particular LPFG, Gaussian curves were the ones presenting the best correlation values.

**Figure 14:**
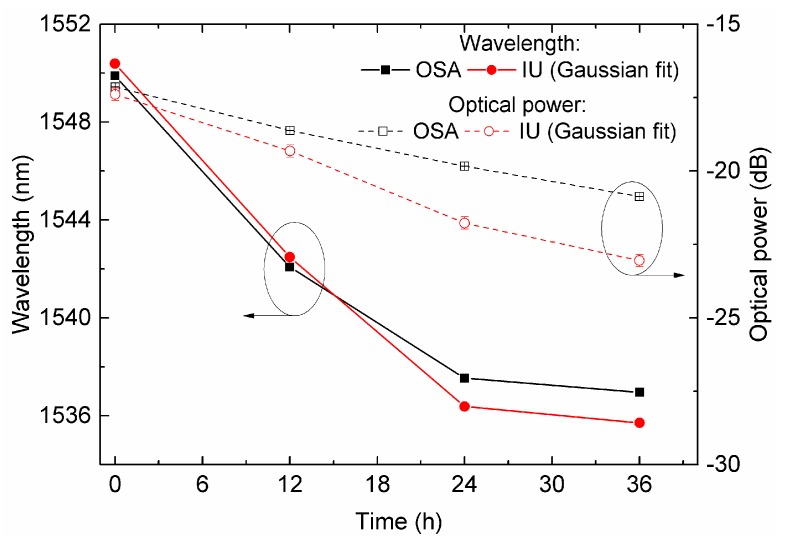
IU measurements for corrosion in salt water of a 20 nm thick Fe-coated LPFG in terms of the peak wavelength and optical power. The measurements obtained by the BBLS/OSA scheme are presented for comparison.

The first measurement revealed values of wavelength and optical power obtained by the IU and measured by the OSA only 0.12 nm and 0.70 dB apart. After 12 h, the grating presented signals of corrosion and the spectral readout revealed a wavelength shift of 8.68 nm and a visibility increase of 1.9 dB. After 24 h, the corrosion shifted the grating peak wavelength and optical power values to 1537.30 nm and 20.17 dB, respectively. After 36 h, the spectra presented a residual increase in optical power of about 0.43 dB, while the wavelength decreased only 0.58 nm, which is expected [19]. A comparison of the corrosion results for the Fe-coated LPFG in terms of optical power and wavelength is presented in Figure 14.

The obtained results, in the region between 1550 and 1535 nm, presented a maximum error of 7.05% in optical power and a difference of 1.12 nm in wavelength.

## 5. Conclusions

A cost-effective and low-power interrogation platform was presented, which can be employed in remote sensing given its data-logging capabilities.

Three thermally modulated fiber-coupled laser diodes, sweeping a few nanometers around their central wavelength positions, and combined with a single photo-detector were shown to be capable of estimating the spectral shape of long-period fiber gratings.

Different curves were fitted by numerical methods to the acquired data, and it was revealed that for each LPFG across several measurements, the same fitting curve type, with different coefficients, presented the best correlation values. It was observed that intrinsic grating asymmetries were the major contribution to the gap between the real spectrum and that obtained by fitting methods. Even with these differences, this technique was capable of tracking LPFG shapes while monitoring different parameters in the range from 1530 nm to 1570 nm within 1.12 nm.

Cascading laser diodes in outer positions, providing more range capability, can be used to increase the measuring range and enhance the obtained fittings, although they will increase the material costs. The developed prototype can be used as a low-cost interrogation technique for remote sensing where only nanometric resolution is needed but long-term measurements are desired.
